# Epidemiological, clinical and radiological characteristics of people with neurocysticercosis in Tanzania–A cross-sectional study

**DOI:** 10.1371/journal.pntd.0010911

**Published:** 2022-11-28

**Authors:** Dominik Stelzle, Charles Makasi, Veronika Schmidt, Chiara Trevisan, Inge van Damme, Tamara M. Welte, Charlotte Ruether, Agnes Fleury, Pierre Dorny, Pascal Magnussen, Gideon Zulu, Kabemba E. Mwape, Emmanuel Bottieau, Sarah Gabriël, Bernard J. Ngowi, Andrea S. Winkler, on behalf of the SOLID collaborators

**Affiliations:** 1 Department of Neurology, Center for Global Health, Faculty of Medicine, Technical University of Munich, Munich, Germany; 2 National Institute for Medical Research, Muhimbili Medical Research Centre, Dar es Salaam, Tanzania; 3 Centre for Global Health, Institute of Health and Society, Faculty of Medicine, University of Oslo, Oslo, Norway; 4 Department of Translational Physiology, Infectiology and Public Health, Faculty of Veterinary Medicine, Ghent University, Ghent, Belgium; 5 Department of Biomedical Sciences, Institute of Tropical Medicine, Antwerp, Belgium; 6 Epilepsy Center, Department of Neurology, University Hospital Erlangen, Erlangen, Germany; 7 Department of Radiology, Faculty of Medicine, Technical University of Munich, Munich, Germany; 8 Departamento de Medicina Genómica y Toxicología ambiental, Instituto de Investigaciones Biomédicas-UNAM / Clínica de neurocisticercosis, Instituto Nacional de Neurología y Neurocirugía, Ciudad de México, Mexico; 9 Faculty of Health and Medical Sciences, University of Copenhagen, Copenhagen, Denmark; 10 Ministry of Health, Lusaka, Zambia; 11 Department of Clinical Studies, School of Veterinary Medicine, University of Zambia, Lusaka, Zambia; 12 Department of Clinical Sciences, Institute of Tropical Medicine, Antwerp, Belgium; 13 University of Dar es Salaam, Mbeya College of Health and Allies Sciences, Mbeya, Tanzania; Fort Collins, UNITED STATES

## Abstract

**Background:**

Neurocysticercosis (NCC) is common among people with epilepsy in low-resource settings. Prevalence of NCC and radiological characteristics of patients with NCC vary considerably even within small areas but differences have been poorly characterized so far.

**Methods:**

We conducted a cross-sectional study between August 2018 and April 2020 in three district hospitals in southern Tanzania (Ifisi, Tukuyu and Vwawa). Patients with and without epileptic seizures were included in this study. All patients were tested with a novel antibody-detecting point-of-care test for the diagnosis of *Taenia solium* cysticercosis. All test positives and a subset of test negatives had a further clinical work-up including medical examination and computed tomography of the brain. NCC was defined according to the Del Brutto criteria. We assessed epidemiological, clinical and radiological characteristics of patients with NCC by presence of epileptic seizures and by serology status.

**Results:**

In all three district hospitals, more than 30% of all people with epileptic seizures (PWE) had NCC lesions in their brain (38% in Vwawa, 32% in Tukuyu and 31% in Ifisi). Most PWE with NCC had multiple lesions and mostly parenchymal lesions (at least 85%). If patients were serologically positive, they had in the median more lesions than serologically negative patients (15 [interquartile range 8–29] versus 5 [1.8–11]), and only serologically positive patients had active stage lesions. Furthermore, serologically positive PWE had more lesions than serologically positive people without epileptic seizures (10.5 [7–23]), and more often had active lesions. PWE diagnosed with NCC (n = 53) were older, and more commonly had focal onset seizures (68% versus 44%, p = 0.03) and headache episodes (34% versus 14%, p = 0.06), which were also stronger than in PWE without NCC (p = 0.04).

**Conclusion:**

NCC is common among PWE. A combination of clinical and serological factors could help to establish an algorithm to identify patients potentially suffering from active NCC, who benefit from further clinical investigation including neuroimaging.

## Author summary

Neurocysticercosis (NCC) is a neglected tropical disease caused by the pork tapeworm *Taenia solium*. The disease remains often silent for years, and some patients never experience any symptoms at all. If the disease becomes symptomatic, symptoms are manifold but most commonly patients suffer from epileptic seizures or headache. Symptoms depend on the location, number and stage of the NCC lesions. Radiological features of NCC differ between world regions and are still understudied for African populations. In this study, we describe radiological but also clinical features of NCC patients with and without epileptic seizures. We found that around one third of patients with epileptic seizures had NCC typical lesions in their brain. Most patients had multiple parenchymal lesions–patients with epileptic seizures had on average more lesions than asymptomatic patients. Patients with epileptic seizures and NCC more commonly had focal onset seizure than patients with epileptic seizures without NCC.

## Introduction

Neurocysticercosis (NCC) is a widely spread neglected tropical disease caused by the pork tapeworm *Taenia solium*. The disease occurs when larvae of this zoonotic parasite (= cysticerci) lodge in the central nervous system. [1] NCC is a public health concern in many low-income and middle-income countries (LMIC), including most countries of sub-Saharan Africa, in which sanitation and hygiene is poor and where pigs roam freely. The disease presents with a variety of different neurological signs and symptoms which depend on the number, stage and location of NCC-typical lesions and on the intensity of the inflammatory reaction. [2] Epileptic seizures are the most common clinical characteristic, especially among patients with lesions in the parenchyma. [3] In areas highly endemic for *T*. *solium* up to one third of acquired epilepsy is caused by the parasite. [4–6] In contrast, patients with lesions in the extraparenchymal space (e.g. in the ventricles) often present with signs of increased intracranial pressure, such as severe progressive headache episodes. To date it is not clear why some cysticerci settle in the parenchyma and others in the extraparenchymal space; reasons include different genotypes of *T*. *solium*, environmental factors and immunological characteristics of the host. This is highlighted by differences in radiological presentation of patients by country/continent. In India, patients often present with epileptic seizures and they commonly have single enhancing lesions in the parenchyma, whereas in Latin America, patients typically experience severe headache and have multiple lesions in the extraparenchymal space. [2,7] Experts consider these two forms as separate disease entities which also require different treatment. Parenchymal lesions can be treated with antiparasitic medication (albendazole and/or praziquantel) whereas for extraparenchymal lesions ventricular shunting or the surgical removal of the cysts is recommended if lesions are located in the ventricles. [8,9] A reason for this varied disease presentation may also be differences in the infection pressure. A study highlighted that with decreasing infection pressure, lesions are mostly found in the extraparenchymal space. [10] Whether this is due to infection pressure or simply because of less specific signs/symptoms and therefore later diagnosis, is still not clear. For Africa, so far only limited information is available on radiological features of NCC which shows African patients to be somewhere in between Indian and Latin American patients, although closer to the latter. [11–13] In other words, African patients mostly had parenchymal lesions, but also mostly multiple lesions. Studies on the genome of *T*. *solium* also revealed the African tapeworm to be closer to the Latin American one. [14–16]

For targeted public health interventions, in-depth knowledge about disease epidemiology is crucial which is why this study aims to evaluate the proportion of patients with NCC among PWE at three different sites in rural southern Tanzania. Furthermore, to facilitate selection for neuroimaging in low-resource settings, the study aims to determine differences in clinical presentation of PWE with and without NCC. Last, to further characterize people with NCC, the study aims to determine the differences in radiological characteristics between study sites, between serologically positive and negative PWE, and between NCC patients with and without epileptic seizures.

## Methods

### Ethics statement

Ethical clearance for the SOLID project was obtained by all study partners: Technical University of Munich, Klinikum rechts der Isar, Ethical Committee (299/18S), the National Ethics Health Research Committee (NatREC) of Tanzania (NIMR/HQ/R.8a/Vol.IX/2597), Institute of Tropical Medicine, Belgium (IRB/AB/ac/112 Ref 1177/17) and the University of Antwerp, Belgium (EC UZA 17/31/352). The SOLID study was registered in the Pan African Trials Registry (PACTR201712002788898). All patients were informed about all parts of the study before inclusion and all patients signed an informed consent. For illiterate patients and for underage patients (<18 years) a guardian signed the informed consent.

### Study details

This study was part of the SOLID project in Tanzania. The main objective of the SOLID project was the evaluation of a novel antibody-detecting lateral-flow point-of-care test (POC test) for the diagnosis of *T*. *solium* taeniosis and (neuro)cysticercosis. The POC test is a lateral flow test which is performed with blood from finger pricking. The test has two strips in a casette for the detection of antibodies–one strip for the diagnosis of *T*. *solium* taeniosis (rES33 protein) and one strip for the diagnosis of (neuro)cysticercosis (rT24H protein). For this, patients with signs/symptoms associated with NCC were recruited from Mental Health Clinics and patients without such signs/symptoms from Out-patient departments. All patients were tested with the POC test and if the test was positive for cysticercosis, the patient received a further clinical work-up including neuroimaging and serological testing (reference tests for the evaluation of the POC test). Also, every tenth POC negative patient from Mental Health Clinics received a clinical work-up. The protocol with the detailed study procedures has been published elsewhere. [17] A visual outline can be found in S1 Fig). The project recruited patients with NCC typical signs/symptoms (epileptic seizures and/or history of severe progressive headache) from the Mental Health Clinic of three district hospitals (Ifisi [Mbeya rural district], Tukuyu [Mbeya rural district] and Vwawa [Mbozi district]) situated in the southern highlands of Tanzania. The screening criteria for these signs/symptoms can be found in the S1 and S2 Tables. For the current study, only data from Mental Health Clinic patients with epileptic seizures were analysed (referred to as people with epileptic seizures [PWE]), regardless of whether they had also screened positive for a history of severe progressive headache. We excluded severe progressive headache patients who did not have a history of epileptic seizures from this analysis because none of the included patients who had a computed tomography (CT) had NCC (n = 17; 4 cysticercosis antibody positive [CC+] and 13 cysticercosis antibody negative [CC-]). Additionally, patients without a history of epileptic seizures or severe progressive headache were recruited from the out-patient departments (OPD) of these district hospitals (referred to as people without epileptic seizures [NPWE]). Further details on the project’s recruitment, study procedures and diagnostic criteria can be found elsewhere. [17]

This study had epidemiological, clinical and radiological objectives. The epidemiological objectives were to determine the prevalence of NCC among PWE by study site and also radiological differences between the study sites. A further objective was the comparison of the clinical characteristics of PWE with and without NCC. Additionally, we assessed radiological characteristics of patients with confirmed NCC. Two types of comparisons were made: a) between serologically positive and serologically negative PWE (sero-status for cysticercosis was determined by the antibody-detecting point-of-care test that was evaluated within the SOLID project); and b) between serologically positive PWE and NPWE. For the second comparison, only serologically positive patients were chosen, because no serologically negative patients from the OPD received a CT scan as in accordance with the study protocol. [17] Last, we also compared clinical characteristics of PWE and NCC by stage of the lesions (at least one active stage lesions versus only calcified lesions).

### Clinical assessment

The clinical work-up consisted of an in-depth history taking, a general medical and neurological examination as well as CT imaging with and without contrast. Data were collected on demography, seizure history and semiology, comorbidities, past medical history, perinatal and family history, and anti-seizure medication. In the neurological examination, we evaluated the patients’ cranial nerves, reflexes, muscle strength, muscle tone, extrapyramidal system, coordination and gait, sensory system, and mental state.

### Radiological assessment and diagnosis of NCC

CT scans were performed at the Mbeya Referral Hospital Radiology Center. The CT scanner used was a Revolution ACT by General Electrics, slice thickness was 1.25mm. CT scans were evaluated by two independent reviewers (CR: neuroradiologist; AF: neurocysticercosis specialist and neurologist) who were blinded to the POC test results. In case of disagreement, a third reviewer (ASW: neurologist) adjudicated the case. We used the neuroimaging criteria defined by Del Brutto et al. for the assessment of patients. [18] The stage of the NCC lesions was categorized as active (viable cysts: vesicular stage; degenerating cysts: colloidal or granular nodular stage), inactive (calcified stage), or mixed (both active and inactive lesions present) stage. [19] The locations of the lesions were classified as parenchymal (frontal lobe, temporal lobe, parietal lobe, occipital lobe, cerebellum, brainstem) or extraparenchymal (intraventricular or subarachnoid).

Diagnosis of NCC was based on the 2017 updated Del Brutto criteria. [18] NCC was classified as definite or probable according to neuroimaging, and clinical/exposure criteria. All PWE included in this analysis were considered to have both minor clinical/exposure criteria fulfilled. NPWE only fulfilled the minor exposure criterion “coming from or living in an area where cysticercosis is endemic”.

### Seizure onset classification

Seizures were classified by type of onset (focal or generalised) according to the 2017 ILAE definition. [20] We based the onset of seizures on semiological characteristics only, because we wanted to use a classification which does not require any additional diagnostic tools as these are rarely available in rural sub-Saharan Africa. If patients reported an aura before seizures, a unilateral seizure onset or an onset of motor signs before loss of consciousness, the seizure was classified as focal onset, otherwise as generalised onset.

### Statistical analyses

The study had two parts. In the first part, the prevalence of NCC among people with epilepsy was determined by study site. We used simulations to account for the study protocol (i.e., every CC+ but only every tenth CC- participant from Mental Health Clinics received further clinical work-up) and loss to follow-up. For this in a first step, the site-specific positive predictive values of the POC test for NCC were determined separately for people recruited for epilepsy only, and for people recruited with a history of epilepsy and additionally a history of severe progressive headache. A single negative predictive value for all sites was determined for all PWE due to the small number of people with negative POC test who had a CT scan. The values are presented in the Supplement (S3 Table). In a second step, these predictive values were multiplied by the number of people, disaggregated by POC test result, site and recruitment reason to determine the estimated number of people with NCC among the recruited PWE. Last, the estimated number of people with NCC was divided by the total number of people recruited to determine the prevalence of NCC among all PWE by site. To account for uncertainty in the data, 10,000 samples following beta-distributions were simulated for the predictive values using the rbeta command. The mean and 95% confidence interval of the 10,000 samples were reported for the estimated number of people with NCC and the proportion of NCC among PWE.

In the second part of the study, confirmed NCC patients were analysed regarding differences in clinical characteristics between PWE and NCC and those without NCC using Chi-square tests for categorical variables and Mann-Whitney U tests for continuous data. Also, comparisons by stage of NCC (mixed stage versus calcified stage; no patient only had active stage lesions) were made using the same methods. Spearman correlation coefficients were used to assess correlations between the total number of (vesicular) NCC lesions and seizure frequency per year. Furthermore, we assessed radiological characteristics between a) serologically positive and negative PWE, and b) serologically positive PWE and NPWE. Mann-Whitney U tests were applied for continuous variables, and Chi-square tests for binary variables.

Numbers and proportions were presented for binary variables; continuous data were presented as the median and interquartile range (IQR). A value of p<0.05 was considered as statistically significant. All analyses were performed using R version 4.1.1.

## Results

**Fig 1:**
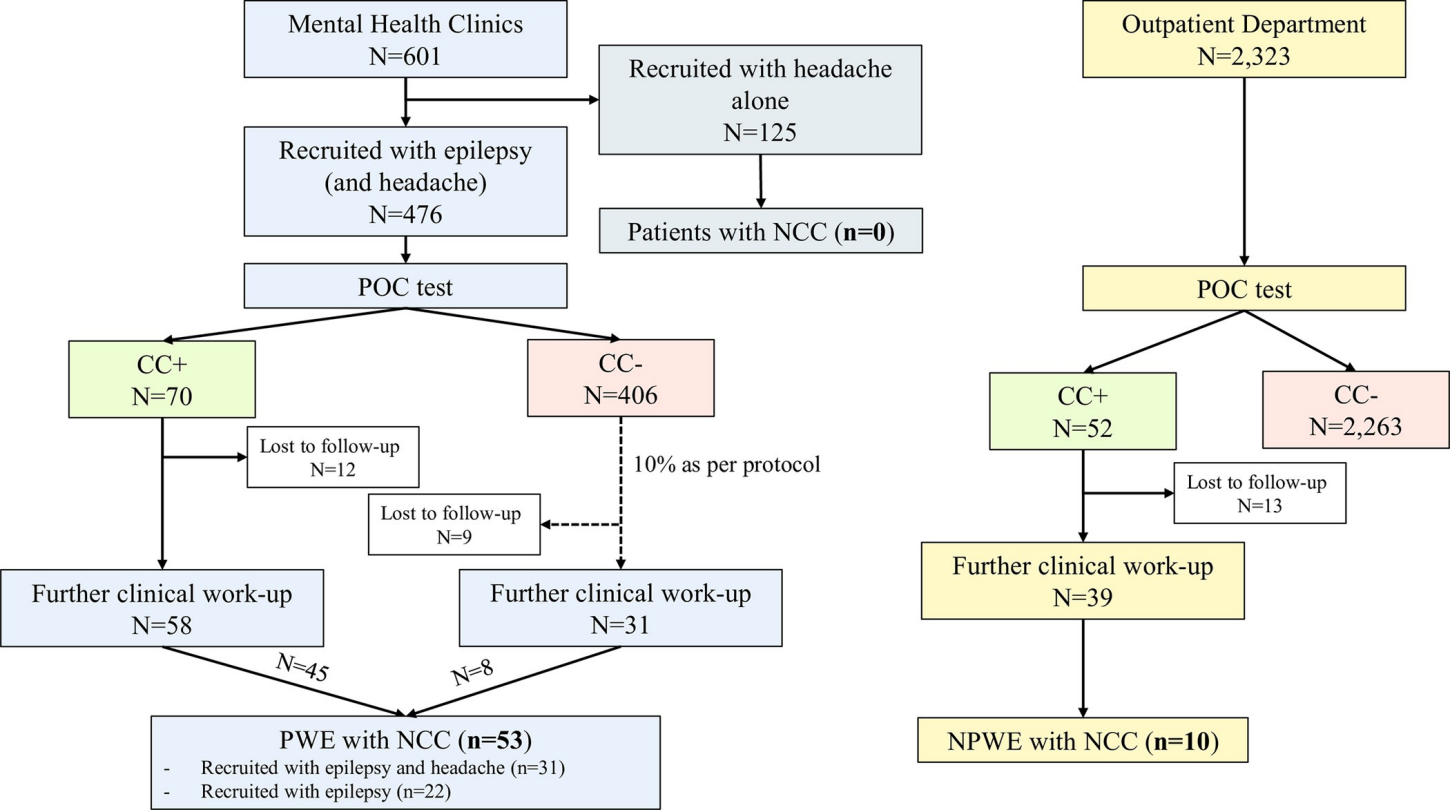
Flowchart of the patients included in this analysis. POC test results include second POC testing. All patients who had their further clinical work-up performed more than 2 months after the first. POC test, were POC tested again on the day of the work-up. The latest POC result is presented here. PWE People with epileptic seizures. NPWE People without epileptic seizures.

Between September 2018 and January 2020, 476 patients with epileptic seizures were included in the SOLID study through mental health clinics (307 of them additionally screened positive for severe progressive headache; Fig 1).

Seventy PWE (15%) had a positive POC test for CC, and 58 (12%) of them had a further clinical work-up. Also, 31 (8%) CC negative PWE had a further clinical work-up. Median age of total 89 PWE with clinical work-up was 36 years (IQR 26 to 50 years). Fifty-five (62%) were male, most were Christians (81, 91%) and peasant farmers (61, 69%). Nearly half of all patients were recruited from Vwawa (41 [46%]; Table 1). Through OPD, 2,323 patients without epileptic seizures (NPWE) were recruited, of which 52 (2.2%) had a positive POC test and 39 (1.7%) had a further clinical work-up. NPWE were in median 48.5 years old (IQR 34 to 58.5 years), 16 (41%) were male, 24 (62%) Christian and 25 (64%) peasant farmers. Most patients were recruited from Vwawa (21, 54%). More details of recruited patients can be found in Table 1.

**Table 1:** Baseline characteristics of people with and without epileptic seizures recruited through the SOLID study.

		Total	Mental Health Clinic (PWE)	Out-patient Department (NPWE)
n		128	89	39
Gender	Female	57 (45)	34 (38)	23 (59)
	Male	71 (55)	55 (62)	16 (41)
Age in years	Median (IQR)	43 [28.5, 54]	36 [26, 50]	48.5 [34, 58.5]
Age group in years	≤20	7 (5)	7 (8)	0 (0)
	21 to 40	56 (44)	40 (45)	16 (41)
	41 to 60	49 (38)	34 (38)	15 (38)
	61 to 80	15 (12)	8 (9)	7 (18)
	>80	1 (1)	0 (0)	1 (3)
Study site	Ifisi	29 (23)	17 (19)	12 (31)
	Tukuyu	37 (29)	31 (35)	6 (15)
	Vwawa	62 (48)	41 (46)	21 (54)
Religion	Christian	105 (82)	81 (91)	24 (62)
	Muslim	4 (3)	1 (1)	3 (8)
	Other or no religion	19 (15)	7 (8)	12 (31)
Tribe	Malila	8 (6)	5 (6)	3 (8)
	Ndali	22 (17)	13 (15)	9 (23)
	Nyakyusa	44 (34)	32 (36)	12 (31)
	Nyha	27 (21)	19 (21)	8 (21)
	Other	27 (21)	20 (22)	7 (18)
Occupation	Peasant farmer	86 (67)	61 (69)	25 (64)
	Professional	9 (7)	6 (7)	3 (8)
	Market/street trader	8 (6)	2 (2)	4 (10)
	Other	27 (21)	20 (22)	7 (18)

PWE People with epileptic seizures

NPWE People without epileptic seizures

MHC Mental Health Clinics

OPD Out-patient department

### Epidemiology of neurocysticercosis among people with epileptic seizures

Fifty-three of 89 PWE had NCC (60%), 9 from Ifisi, 14 from Tukuyu and 30 from Vwawa. Twenty-nine of 53 (55%) NCC patients had active or mixed (active and inactive) stage lesions. Among the 58 patients with positive POC test, 45 (78%) had NCC and among the 31 with negative POC test, 8 (26%) had NCC. Furthermore, the likelihood of NCC varied by site and recruitment reason. The positive predictive value of the POC test was 33.3%, 100% and 80.7% for people recruited with epilepsy and additional severe headache in Ifisi, Tukuyu and Vwawa, respectively. For people recruited with epilepsy only, PPV were 62.5%, 87.5% and 100% at the three sites. The NPV across sites was 28.6% for PWE and severe headache, and 23.5% for PWE without severe headache (S3 Table). Applying these PPV and NPV to the study population yielded NCC prevalence proportions among PWE of 31%, 32% and 38% for Ifisi, Tukuyu and Vwawa (Table 2). Accordingly, the total number of lesions and the total number of active lesions were lowest in Ifisi and highest in Vwawa. Also, POC positive patients had more lesions than POC negative patients (p<0.001), and only POC positive patients had active stage lesions. The proportion of patients with active stage NCC was higher in Tukuyu (72%) and Vwawa (67%), than in Ifisi (43%). The proportion of extraparenchymal lesions among all active stage lesions was highest in Ifisi (2/3, 67%) and lowest in Vwawa (75/356, 21%).

**Table 2:** Neurocysticercosis prevalence among people with epileptic seizures and radiological characteristics of neurocysticercosis by study site.

Study site	Total		Ifisi		Tukuyu		Vwawa	
People with epileptic seizures recruited	476		86		151		239	
NCC cases observed	53		9		14		30	
NCC cases estimated by extrapolation (95%CI)	165 (146–184)		27 (22–32)		48 (41–55)		91 (80–101)	
NCC prevalence among PWE (95%CI)	35% (31–39%)		31% (25–37%)		32% (27–36%)		38% (34–42%)	
POC test result	Negative	Positive	Negative	Positive	Negative	Positive	Negative	Positive
Number of patients with NCC	8	45	2	7	3	11	3	27
NCC-typical lesions								
Number	82	1012	40	88	9	159	33	765
Mean per patient	10.3	22.4	20	12.6	3	14.5	11	28.3
Median per patient [IQR]	5 [1.75–11]	15 [8–29]	20 [12–28]	4 [2–20.5]	2 [1.5–4]	13 [10–19.5]	6 [3.5–16]	25 [9–35]
Active NCC-typical lesions								
Number of patients with active lesions (% of all patients with NCC)	0	29/45 (64%)	0	3/7 (43%)	0	8/11 (72%)	0	18/27 (67%)
Number of lesions	0	402	0	3	0	37	0	362
Mean per patient	0	8.8	0	0.4	0	3.4	0	13.4
Median per patient [IQR]	0	1 [0–1]	0	0 [0–1]	0	4 [0.5–5]	0	5 [3–11]
Active lesions among all lesions (%)		39%		3%		23%		47%
Median proportion of active lesions per patient (%) [IQR]		14% [0–45%]		0% [0–3%]		18% [4–35%]		33% [0–67%]
Location of active lesions (parenchymal/extraparenchymal)								
Parenchymal (N)		304		1		22		281
Extraparenchymal (N)		98		2		15		81
Extraparenchymal lesions (%)		24%		67%		43%		22%

PWE People with epileptic seizures

NCC Neurocysticercosis

IQR Interquartile range

N Number

95%CI 95% confidence interval

⸸ Extrapolation used the site-and recruitment-specific PPV for NCC if POC test was positive; for POC negative patients a recruitment-specific NPV combined for all three sites was used.

### Clinical characteristics of people with epileptic seizures with and without neurocysticercosis

89 PWE received a CT scan of which 53 had NCC (Table 3). Patients with NCC were more commonly female (25/53 [47%] among NCC patients and 9/36 [25%] among patients without NCC, p = 0.06). Patients with NCC were in the median older than patients without NCC (44 years versus 29 years, p = 0.01), and the age of seizure onset was in the median six years later, albeit this was statistically not significant (28 versus 22 years, p = 0.50). Onset of seizures was more commonly focal in NCC patients, an aura was more common among PWE and NCC (62% versus 31%, p = 0.02) and movement was less often bilateral (87% versus 97%, p = 0.02). There were no differences in seizure frequency between both groups either before ASM initiation or after ASM initiation. The decrease of seizure frequency did not differ between both groups either. Phenobarbitone was the most prescribed ASM. Four of the 53 NCC patients (9%) had mental health disorders, and 6 of 36 (17%) patients without NCC. Mostly, these disorders were psychotic episodes. Seven (13%) NCC patients and one patient without NCC (3%) were living with HIV (p = 0.17). NCC patients more commonly had regular headache episodes than patients without NCC (18/53 [34%] versus 5/36 [14%], p = 0.06) and headache was on average reported to be stronger (p = 0.02). There were no differences in the duration of headache, but episodes occurred more frequently among patients with NCC and had more of a throbbing character; fifty percent of patients with NCC and regular headache reported up to weekly episodes compared with only 20% of patients without NCC. Neurological deficits were rare, but, if present, were most commonly cranial nerve deficits (6/83, 7%). However, there were no differences between PWE with and without NCC.

**Table 3:** Demographic and clinical characteristics of people with epileptic seizures with and without neurocysticercosis.

		Overall	NCC	No NCC	p-value
N		89	53	36	
Site	Ifisi	17 (19)	9 (17)	8 (22)	0.05
	Tukuyu	31 (35)	14 (26)	17 (47)	
	Vwawa	41 (46)	30 (57)	11 (31)	
Sex	Female	34 (38)	25 (47)	9 (25)	0.06
	Male	55 (62)	28 (53)	27 (75)	
Age	Median in years	36 [26, 50]	44 [32, 52]	29 [21.75, 48.5]	0.01
Age group in years	(0,20]	7 (8)	1 (2)	6 (17)	<0.001
	(20,40]	40 (45)	20 (38)	20 (56)	
	(40,60]	34 (38)	29 (55)	5 (14)	
	(60,80]	8 (9)	3 (6)	5 (14)	
Religion	Christian	81 (91)	46 (87)	35 (97)	0.04
	Muslim	1 (1)	0 (0)	1 (3)	
	Other or no religion	7 (8)	7 (13)	0	
Age of seizure onset (years)	Median [IQR]	27 [17, 41.25]	28 [19.5, 38]	22 [15.5, 47.5]	0.50
Time since seizure onset (years)	Median [IQR]	9 [3.25, 16]	13 [6, 20.75]	5.5 [2.25, 11.75]	<0.001
Motor activity during seizures	Tonic-clonic	65 (74)	38 (73)	27 (75)	0.61
	Clonic	4 (5)	3 (6)	1 (3)	
	Tonic	6 (7)	2 (4)	4 (11)	
	Myoclonic (short, jerky movement)	1 (1)	1 (2)	0 (0)	
	No movement of limbs, but rolling of eyes and grinding of teeth	7 (8)	4 (8)	3 (8)	
Focal onset of seizures		52 (58)	36 (68)	16 (44)	0.03
Side of onset of seizures	Bilateral	68 (86)	36 (87)	32 (97)	0.04
	Unilateral	11 (14)	10 (21)	1 (3)	
	Not applicable	10	7	3	
Loss of consciousness	No loss of consciousness	1 (1)	1 (2)	0 (0)	0.69
	Yes, after motor signs start	16 (18)	9 (17)	7 (19)	
	Yes, from the beginning	72 (81)	43 (81)	29 (81)	
Aura		44 (50)	33 (62)	11 (31)	0.02
Seizure frequency before treatment	Daily to monthly	30 (35)	17 (33)	13 (38)	0.23
	Monthly to yearly	43 (50)	28 (54)	15 (44)	
	Yearly	3 (4)	3 (6)	0	
	Less than yearly, irregularly	10 (12)	4 (8)	6 (18)	
	Not available	3	1	2	
	Median [IQR]	12 [6, 24]	12 [6, 24]	12 [6, 24]	0.84
Seizure frequency after treatment	Daily to monthly	4 (5)	2 (4)	2 (6)	0.80
	Monthly to yearly	32 (37)	20 (39)	12 (35)	
	Yearly	16 (19)	11 (21)	5 (15)	
	Less than yearly, irregularly	34 (40)	19 (37)	15 (44)	
	Not available	3	1	2	
	Median [IQR]	1 [0.3, 4]	1 [0.5, 4]	1 [0.14, 5.5]	0.72
**Risk factors**					
Illness before first seizure		7 (8)	3 (6)	4 (11)	0.52
Head trauma		3 (3)	2 (4)	1 (3)	0.99
Birth/childhood history	Complications during birth⸸	3 (3)	2 (4)	1 (3)	0.99
	Milestones delayed	4 (4)	1 (2)	3 (8)	
Febrile seizures history		9 (10)	5 (9)	4 (11)	0.99
Family	Family member with epilepsy	17 (19)	11 (21)	6 (17)	
	*First degree*	*13/17 (76)*	*8/11 (73)*	*5/6 (83)*	
	*Second degree*	*4/17 (24)*	*3/11 (27)*	*1/6 (17)*	
**Treatment**					
Anti-seizure medication (ASM)	Currently on ASM	74 (83)	46 (87)	28 (78)	0.29
	*Monotherapy*	*42/74 (57)*	*23/46 (50)*	*19/28 (68)*	0.07
	*Combination therapy*	*32/74 (43)*	*23/46 (50)*	*9/28 (32)*	
	Phenobarbitone	64 (86)	39 (85)	25 (89)	
	Phenytoin	9 (12)	8 (17)	1 (4)	
	Carbamazepine	33 (45)	22 (48)	11 (39)	
Herbal treatment		42 (47)	26 (49)	16 (44)	0.77
Scarification		16 (18)	6 (11)	10 (28)	0.10
**Impact of seizures**					
Injuries		30 (34)	14 (26)	16 (44)	0.14
Schooling		66 (74)	42 (79)	24 (67)	0.99
Drop-out from school		13 (15)	8 (15)	5 (14)	0.99
**Chronic diseases**					
Mental health disorders	Yes	10 (11)	4 (9)	6 (17)	0.49
	Psychotic episodes	7/10 (70)	3/4 (75)	4/6 (67)	
	Dementia/memory loss	1/10 (10)	0	1/6 (17)	
	Other or NA	2/10 (20)	1 (25)	1/6 (17)	
	*Mild*	*5/10 (50)*	*3/4*	*2/5*	
	*Moderate*	*1/10 (10)*	*0*	*1/5*	
	*Severe*	*3/10 (30)*	*1/4*	*2/5*	
HIV infection		8 (9)	7 (13)	1 (3)	0.17
**Headache history**					
Frequent history of headache		23 (26)	18 (34)	5 (14)	0.06
Headache severity	0 = Almost no hurt	1 (4)	1 (2)	0 (0)	0.04
	1 = Hurts just a little bit	4 (17)	1 (2)	3 (8)	
	2 = Hurts a little more	4 (17)	4 (8)	0 (0)	
	3 = Hurts even more	7 (30)	5 (9)	2 (6)	
	4 = Hurts a whole lot	7 (30)	7 (13)	0 (0)	
	5 = Worst pain imaginable	0 (0)	0 (0)	0 (0)	
Headache quality	pressure	3 (13)	3 (6)	0 (0)	0.39
	piercing	2 (9)	1 (2)	1 (3)	0.99
	throbbing	18 (78)	15 (28)	3 (8)	0.04
	stabbing	4 (17)	2 (4)	2 (6)	0.99
Headache duration	Up to one hour	11 (48)	9 (50)	2 (40)	0.31
	Between one hour and one day	8 (35)	5 (28)	3 (60)	
	One day or longer	4 (17)	4 (22)	0	
Headache frequency	Up to weekly	10 (43)	9 (50)	1 (20)	0.02
	weekly to monthly	11 (48)	9 (50)	2 (40)	
	Less than once per month	2 (9)	0	2 (40)	
**Neurological examination**					
Cranial nerve deficit		6 (7)	2 (4)	4 (11)	0.24
Muscle atrophy		2 (2)	1 (2)	1 (3)	0.57
Muscle strength deficit		1 (1)	1 (2)	0	0.25
Extrapyramidal system abnormalities		3 (3)	1 (2)	2 (6)	0.45
Deep tendon reflexes increased		1 (1)	1 (2)	0 (0)	0.25
Primitive reflexes present		0	0	0	NA
Sensory deficit		1 (1)	0	1 (3)	0.48
Abnormal coordination/gait		4 (4)	2 (4)	2 (6)	0.92

NCC Neurocysticercosis; IQR Interquartile range; ASM Anti-seizure medication

⸸ Pre-term birth or prolonged labour

### Radiological and clinical characteristics of people with neurocysticercosis

All 53 PWE with NCC (CC+ and CC-) had a definite diagnosis according to the Del Brutto criteria. The 45 CC+ PWE had in total 1,012 lesions of which 402 were in active stage; the 8 CC- PWE had in total 82 lesions of which none was in active stage (Table 4). Serologically positive PWE had in the median more lesions than serologically negative PWE (15 lesions [IQR 8 to 29] versus 5 [1.8 to 11], p<0.001). The 10 CC+ NPWE had overall 171 lesions of which 76 were in active stage. Nine of these 10 (90%) NPWE had a definite NCC diagnosis. Serologically positive NPWE had in the median 10.5 lesions [7 to 23], fewer than serologically positive PWE (p<0.001). Only serologically positive patients (PWE and NPWE) had active stage lesions: PWE on average 8.9 (median = 1) and NPWE on average 7.6 (median = 1). Almost 90% of all lesions were in the brain parenchyma–mostly the telencephalon. In total, 27 lesions (2.1%) were in the cerebellum and five (0.4%) in the brainstem. Almost all lesions in the extraparenchymal space were located in the subarachnoid space around the cortex; only eight (0.6%) lesions were found in the ventricles.

**Table 4:** Location of cysts among people with epileptic seizures and those without epileptic seizures.

		PWE POC CC+ (n = 45) N (%)	PWE POC CC- (n = 8) N (%)	NPWE POC CC+ (n = 10) N (%)
Certainty of diagnosis	Definite	45 (100)	8 (100)	9 (90)
	Probable	0	0	1 (10)^⸸^
All patients	Number	45	8	10
	Number of lesions	1012	82	171
	Mean per patient	22.5	10.3	17.1
	Median per patient [IQR]	15 [8–29]	5 [1.8–11]	10.5 [7–23]
Patients with active lesions	Number of patients	29/45 (64)	0/8 (0)	6/10 (60)
	Number of lesions	402	0	76
	Mean per patient	8.9	0	7.6
	Median per patient [IQR]^£^	1 [0–9]	0 [0–0]	1 [0–4]
Patients with calcifications	Number of patients	45/45 (100)	8/8 (100)	10/10 (100)
	Number of lesions	610	82	95
	Mean per patient	13.6	10.3	9.5
	Median per patient [IQR]	9 [6–18]	5 [1.8–11]	8 [4–12.3]
Location of lesions	Parenchymal	860 (85)	72 (88)	148 (87)
	Extra-parenchymal	152 (15)	10 (12)	23 (13)
Parenchymal	Frontal	408 (40)	28 (34)	78 (46)
	Temporal	172 (17)	14 (17)	23 (13)
	Parietal	116 (11)	10 (12)	19 (11)
	Occipital	140 (14)	19 (23)	21 (12)
	Cerebellum	20 (2)	1 (1)	6 (4)
	Brainstem	4 (0)	0 (0)	1 (1)
Extra-parenchymal	Sub-arachnoid	144 (14)	10 (12)	23 (13)
	Intra-ventricular	8 (1)	0 (0)	0 (0)

NCC Neurocysticercosis; IQR Interquartile range

⸸ According to the Del Brutto criteria, only the following reference tests but not the POC test were considered for diagnosis: LLGP-EITB, rT24H-EITB and antigen ELISA. The one patient with probable diagnosis was negative in all three tests.£ Mann-Whitney U test: PWE POC CC+ versus CC-: p<0.001; PWE CC+ versus NPWE CC+: p<0.001

There were no marked differences in clinical characteristics between patients with mixed stage NCC and only calcified stage NCC (S4 Table). Patients with only calcified lesions had experienced epileptic seizures in the median 6 years longer than patients with mixed stage NCC (15 years versus 9 years, p = 0.67). The total number of (vesicular) NCC lesions and the frequency of epileptic seizures per year were positively correlated; the correlation was stronger for vesicular lesions (Spearman rho = 0.28) than for any kind of NCC lesions (Spearman rho = 0.09); however, neither correlation was strong or statistically significant (p = 0.54, and p = 0.28; S2 Fig).

## Discussion

In this study, we assessed NCC prevalence among PWE in three districts in the southern highlands of Tanzania. We furthermore assessed clinical characteristics of PWE with and without NCC. Last, we also analysed clinical and radiological differences of patients with confirmed NCC according to serological screening, presence of seizures, and by the stage of NCC.

We found that in all three district hospitals, more than 30% percent of all PWE had NCC-typical lesions in their brain. We found that clinical characteristics of PWE with NCC differed in some respects from PWE without NCC; they more commonly had focal onset seizures and more commonly had headache episodes which were also stronger. Also, we found that most patients with NCC had multiple lesions and mostly parenchymal lesions. If patients were serologically positive, they had on average more lesions than serologically negative patients, and only serologically positive patients had active stage lesions. Furthermore, PWE had more lesions than NPWE. Clinical characteristics did not differ by the stage of NCC but seizure frequency was stronger correlated with the total number of active lesions than with any kind of lesions.

Our assessment of the proportion of PWE and NCC yielded similar results as previously published; around one third of PWE had NCC-typical lesions. However, prevalence proportions differ substantially even within small areas (e.g., on a village level) due to variation in the distribution of risk factors such as pigs roaming freely, poor sanitation and hygiene. Whether epilepsy is due to these lesions is not clear as some of these patients may have developed epilepsy even without NCC. In our study, in Mbeya rural district (Ifisi and Tukuyu) fewer PWE had NCC than in Mbozi district (Vwawa). This could be due to a higher infection pressure for *T*. *solium* in the latter. This is also underpinned by the fact that a higher proportion of patients from Mbozi district had active stage lesions, that they had in the median more lesions and that proportionally more lesions were in the parenchyma. This association between disease phenotype and infection pressure has been hypothesized previously by Hamamoto et al. who described a proportional increase in extraparenchymal lesions with a decreasing infection pressure. [10]

These differences in radiological presentation of NCC between sites are also in line with a previously published paper on porcine CC which found a significantly higher prevalence at carcase dissection in Mbozi district (22/154; prevalence 14.3%, 95%CI 9.2 to 20.8%) compared with Mbeya rural district (4/128, prevalence 3.1%, 95%CI 0.9 to 7.8%). [21] Also, in Mbozi district more pigs were free ranging (26.5% versus 10.5%), and more pigs had access to latrines according to the pig farmers. Likewise, risk awareness was lower in Mbozi district than in Mbeya rural district where only 3.1% of pig farmers (compared with 9.5%) considered porcine cysticercosis as a public health problem. As the life cycle of *T*. *solium* requires both humans and pigs, endemicity of *T*. *solium* cysticercosis in pigs can be used as a proxy for human cysticercosis. Due to the shorter life expectancy of pigs, porcine CC may reflect changes in the infection pressure more quickly and the examination of pigs/pork may therefore more effectively monitor disease burden than examination of humans. Interestingly, Christian PWE did not show a higher proportion of NCC than Muslim PWE or PWE of other faith, highlighting that transmission does not necessarily depend on people themselves eating pork or living with pigs but that in endemic areas contamination of food and soil might also be a driving force. [22] However, there were only very few Muslims and people with other faith in our study.

In this study, we also assessed detailed clinical characteristics of PWE with NCC compared to PWE without NCC. Our main finding was, that PWE with NCC more often revealed signs of focal seizure onset and frequently reported aura. This is in line with several other studies and explained by the underlying pathophysiology. [5,23,24]

PWE with NCC were older compared to PWE without NCC, which has been described previously, as NCC often manifests clinically only after several years. [23,25] Seen the other way round, this finding also could indicate that non-NCC associated epilepsies in these areas might have aetiologies usually manifesting in childhood or early adulthood, e.g. generalized epilepsies. The fact that non-NCC PWE also more often showed a generalized seizure onset could support that. This is in line with other studies arguing that not only focal, acquired, infectious epilepsies are responsible for the high burden of epilepsy in SSA but genetic factors may also play a role. [26,27]

In terms of anti-seizure treatment, similar to many studies in SSA settings, most common administered ASM were phenobarbitone and carbamazepine, and a large proportion of PWE in both groups also were treated by traditional healers including herbal treatment and scarification. [28–30] After administration of regular ASM, there was a significant drop in seizure frequency in both groups, indicating a good treatment response in both NCC and non-NCC related epilepsies but also underlining the need for optimization of ASM therapy in PWE populations that still lack uninterrupted medication supply and specialized healthcare personnel for e.g. adequate dosage advice. [28,30–32] These circumstances are also reflected by the persisting constraints to regular schooling for PWE as indicated in our results.

In detailed treatment analysis PWE with NCC received more often a combination of ASM than PWE without NCC, which might indicate difficulties in seizure treatment. Moreover, patients with mixed lesions showed a higher persistence of seizures despite adequate ASM treatment compared to inactive stage NCC, probably indicating treatment difficulties in active NCC. [25] These findings are not in line with previous results from our own group indicating that PWE and NCC had a better response to ASM than PWE without NCC, emphasizing the need for further investigations. [11]

Besides epileptic seizures, headaches are a common symptom of NCC. In the SOLID study none of the patients with severe progressive headache alone had NCC on neuroimaging. Our findings suggest that the additional presence of severe headache in PWE could be another important feature leading to the suspicion of a NCC diagnosis. The type of headache was mostly described as throbbing, which is typical for migraine-like headache. Although detailed information for classification is missing (e.g., unilaterality and accompanying symptoms), this finding is confirmed by a previous study which found a high proportion of patients suffering from migraine-like headache especially if calcified lesions were present. [33]

### Strengths and limitations

Our study had several strengths. To our knowledge, this is one of the largest studies from Africa that assessed detailed radiological and clinical characteristics of patients with NCC. The detailed radiological assessment allowed us to differentiate clinical characteristics of NCC patients by stage of the diseases. This is helpful for therapeutic decisions as only patients with active stage disease require treatment with antiparasitic medication. Another strength was our assessment of serologically positive and negative patients which is often overlooked, as often only serologically positive patients receive neuroimaging. We were able to demonstrate that also serologically negative patients can have NCC, but in our case only in inactive stage and with fewer lesions than serologically positive patients. A further strength was that we included patients from three districts with and without NCC-typical signs/symptoms and patients were recruited during the same period and using the same questionnaires. This allowed for an unbiased assessment of differences between symptomatic and asymptomatic patients, and it also allowed us to compare prevalence of NCC by study site.

However, our study had limitations, too. One limitation was that between inclusion in the study and the CT scan, sometimes several months had passed which is why we tested patients again with the POC test on the day of the CT scan. However, on that day we did not take blood sample again for reference test analysis. That resulted in us only being able to compare radiological characteristics by sero-status according to the POC test. This may have affected our results but as the POC test showed very good accuracy for the diagnosis of NCC we do not believe our results were biased considerably. Currently, the POC test is not available to countries but hopefully after successful evaluation of its accuracy and cost-effectiveness, it will be endorsed by World Health Organization. This would be a huge step towards better monitoring of NCC in resource-limited countries.

Another limitation was the assessment of severe progressive headache. At inclusion, patients were screened for severe progressive headache with a questionnaire that was designed by us as there is no standardised questionnaire for the detection of NCC-typical headache. The evaluation of this screening questionnaire yielded vastly different results by study site likely because questions are rather unspecific. For that reason and because nobody of those patients who received a CT scan had NCC, we excluded these patients from the analysis. Furthermore, our analysis of clinical characteristics of PWE is not comparable to studies in high-income countries, where electro-encephalogram and magnetic resonance imaging are commonly available allowing for a far more accurate diagnosis of epilepsy syndromes. In our cohort we only had CT scan available, and for determination of seizure onset we relied on semiology ascertained through history only, as this is the most common situation in LMIC. Furthermore, by comparing PWE with and without NCC, we analysed serologically positive and negative patients together although selection for further clinical work-up depended on sero-status according to the study design. This may have affected our results, but we do not think the effect was large. Neurological characteristics of PWE without NCC likely do not differ considerably between serologically positive and negative patients as serological tests are cysticercosis- and not neurocysticercosis-specific. That means, these positive patients without NCC lesions probably suffer from cysticercosis.

### Conclusion

We demonstrated that NCC is common among PWE in Mbeya rural and Mbozi district in southern Tanzania. We also showed differences in NCC epidemiology and presentation are likely due to differences in the infection pressure which can be linked to presence of risk factors and absence of risk awareness. Infection pressure also seems to have an impact on the prevailing neuroradiological phenotype of NCC. Overall, our data suggest that in endemic areas, PWE in mid-adulthood who show indicators for focal seizure semiology and especially those with seizure onset in adulthood, have the highest risk of suffering from NCC. These patients in particular may benefit from further clinical investigation and neuroimaging. If in addition severe headache is present, diagnosis of NCC is even more likely. The establishment of a diagnostic algorithm for low-resource settings is crucial to pre-select patients who may benefit from neuroimaging because they most likely suffer from active NCC and may need antiparasitic treatment.

## Supporting information

S1 Fig Study procedure of the SOLID project in Tanzania. (DOCX)

S2 Fig Correlation between total number of (vesicular) NCC lesions and seizure frequency per year (A total number of NCC lesions, B total number of vesicular NCC lesions). (DOCX)

S1 Table Screening questionnaire for epileptic seizures and severe progressive headache. (DOCX)

S2 Table Screening questionnaire for severe progressive headache. (DOCX)

S3 Table Predictive values by site and disaggregated by recruitment reason. (DOCX)

S4 Table Demographic and clinical characteristics of patients with mixed stage and inactive stage neurocysticercosis. (DOCX)
