# Supplementary material for: Epidemiological, clinical and radiological characteristics of people with neurocysticercosis in Tanzania–A cross-sectional study
Source: PLoS Negl Trop Dis. 2022 Nov 28;16(11):e0010911. doi: 10.1371/journal.pntd.0010911 (PMC9704569; doi:10.1371/journal.pntd.0010911)
Supplement: S1 Fig — (DOCX) [file pntd.0010911.s001.docx]

S1 Fig. Study procedure of the SOLID project in Tanzania


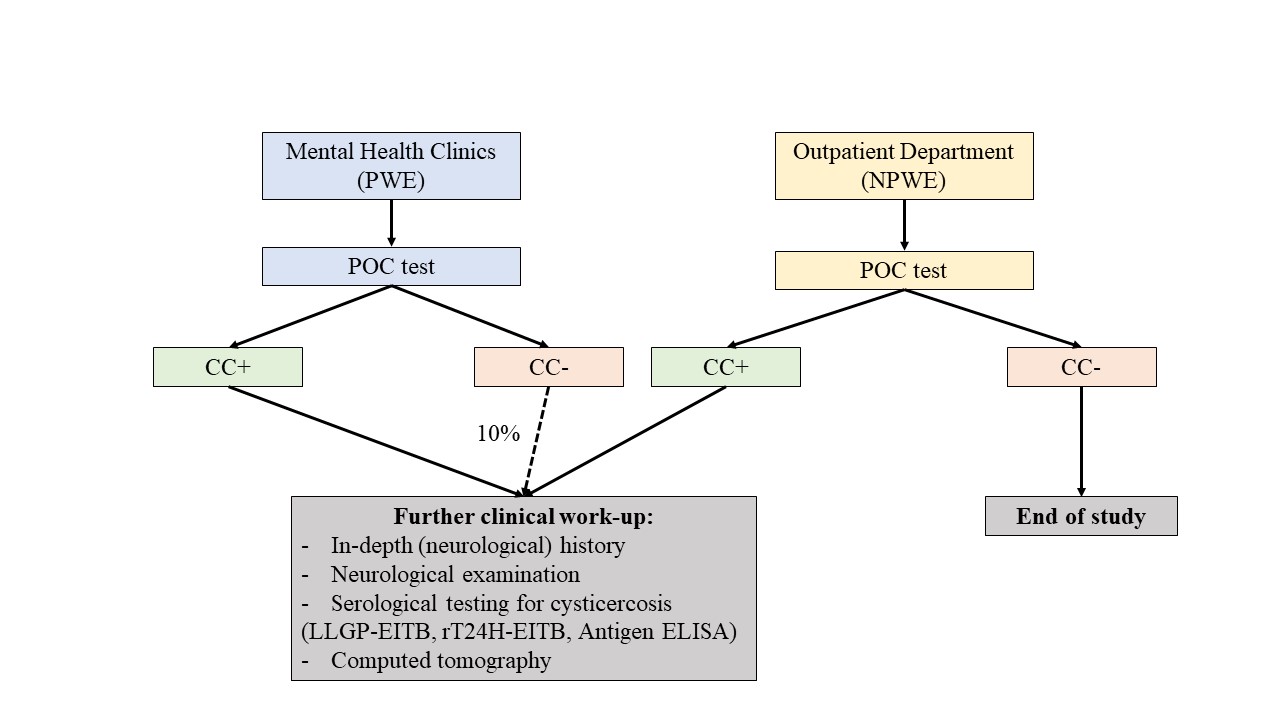
POC test: point-of-care test; CC+: cysticercosis positive; CC-: cysticercosis negative
