# Supplementary material for: Epidemiological, clinical and radiological characteristics of people with neurocysticercosis in Tanzania–A cross-sectional study
Source: PLoS Negl Trop Dis. 2022 Nov 28;16(11):e0010911. doi: 10.1371/journal.pntd.0010911 (PMC9704569; doi:10.1371/journal.pntd.0010911)

S2 Fig. Correlation between total number of (vesicular) NCC lesions and seizure frequency per year (A total number of NCC lesions, B total number of vesicular NCC lesions)


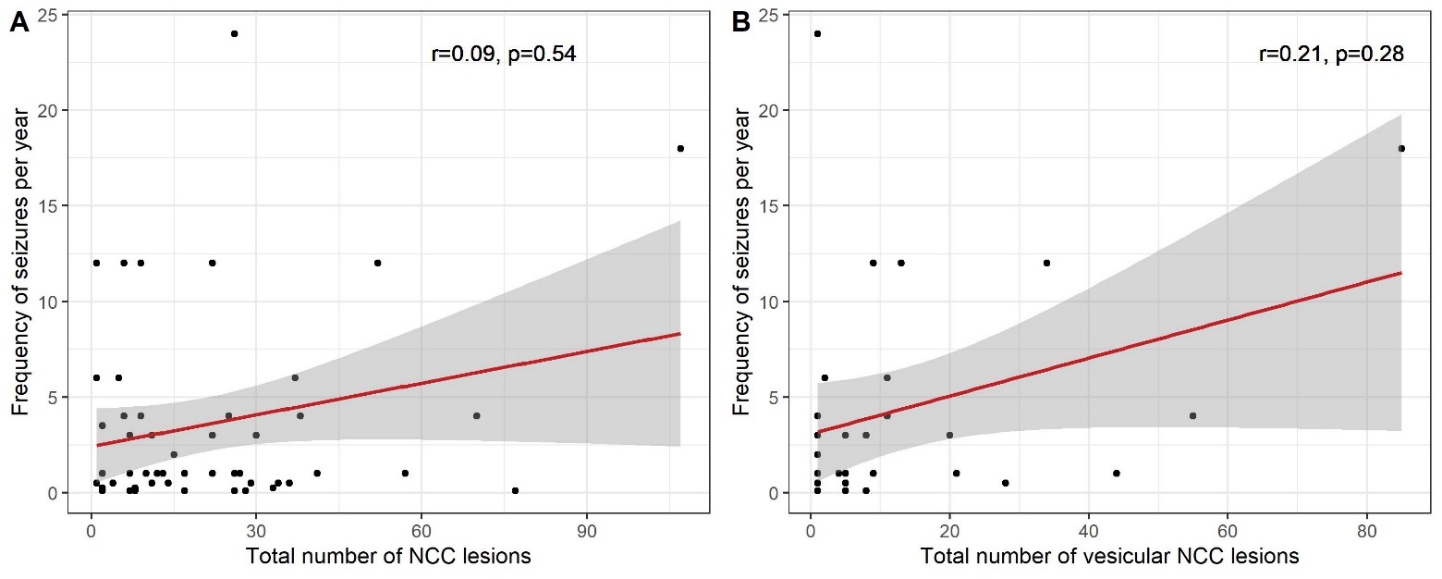

Supplement: S2 Fig — (DOCX) [file pntd.0010911.s002.docx]
