# Supplementary material for: Epidemiological, clinical and radiological characteristics of people with neurocysticercosis in Tanzania–A cross-sectional study
Source: PLoS Negl Trop Dis. 2022 Nov 28;16(11):e0010911. doi: 10.1371/journal.pntd.0010911 (PMC9704569; doi:10.1371/journal.pntd.0010911)
Supplement: S1 Table — (DOCX) [file pntd.0010911.s003.docx]

S1 Table. Screening questionnaire for epileptic seizures and severe progressive headache

| **N°** | **Question** | **Comment** |
| --- | --- | --- |
| **Screening questionnaire for epileptic seizures** | | |
| 1 | Have you ever been told that you had an episode of losing consciousness during which your arms and legs shake or stretch out? | Yes / No / Do not know |
| 2 | During attacks of unconsciousness have you ever bitten your tongue or lost control of your bladder or bowels? | Yes / No / Do not know |
| 3 | Have you ever had uncontrollable attacks of shaking or trembling in one arm or one leg or in the face without losing consciousness? | Yes / No / Do not know |
| 4 | Have you ever been told that you have or had epilepsy or epileptic seizures? | Yes / No / Do not know |
|  | *Only* ***one Yes*** *is required to screen positive for epileptic seizures.* | Screen positive / Screen negative |
