# Supplementary material for: Epidemiological, clinical and radiological characteristics of people with neurocysticercosis in Tanzania–A cross-sectional study
Source: PLoS Negl Trop Dis. 2022 Nov 28;16(11):e0010911. doi: 10.1371/journal.pntd.0010911 (PMC9704569; doi:10.1371/journal.pntd.0010911)
Supplement: S2 Table — (DOCX) [file pntd.0010911.s004.docx]

S2 Table. Screening questionnaire for severe progressive headache

| **N°** | **Question** | **Comment** |
| --- | --- | --- |
| **Screening questionnaire for severe progressive headache** | | |
| 1 | Have you recently (within the past one month including now) suffered from severe headaches? | Yes / No / Do not know |
| 2 | Are/were those headaches keeping you from performing your work or usual activities? | Yes / No / Do not know |
| 3 | Are/were those headaches increasing progressively over time? | Yes / No / Do not know |
| 4 | Do you normally suffer from headaches? | Yes / No / Do not know |
| 5 | Are/were your recent headaches different from your usual headaches? | Yes / No / Do not know |
|  | *If* ***1, 2 and 3*** *are* ***Yes****, or if* ***4*** ***and 5*** *are* ***Yes****, the person is screen positive for severe progressive headache.* | Screen positive / Screen negative |
