# Supplementary material for: Epidemiological, clinical and radiological characteristics of people with neurocysticercosis in Tanzania–A cross-sectional study
Source: PLoS Negl Trop Dis. 2022 Nov 28;16(11):e0010911. doi: 10.1371/journal.pntd.0010911 (PMC9704569; doi:10.1371/journal.pntd.0010911)
Supplement: S3 Table — (DOCX) [file pntd.0010911.s005.docx]

S3 Table. Predictive values by site and disaggregated by recruitment reason

|  | **Predictive values** | **Ifisi** | **Tukuyu** | **Vwawa** |
| --- | --- | --- | --- | --- |
| Epilepsy and Headache | PPV | 33.3% (SD 17.8%) | 99% (SD 1%)⸸ | 80.7% (SD 7%) |
|  | NPV | 28.6% (SD 2.8%) | 28.6% (SD 2.8%) | 28.6% (SD 2.8%) |
| Epilepsy only | PPV | 62.5% (SD 16.1%) | 87.5% (SD 11%) | 99% (SD 1%)⸸ |
|  | NPV | 23.5% (SD 3.5%) | 23.5% (SD 3.5%) | 23.5% (SD 3.5%) |

PPV positive predictive value

NPV negative predictive value

SD standard deviation

⸸ In the dataset, 100% had NCC. The value was changed to account for variance in this finding.

10,000 samples of beta-distributions of these predictive values were simulated using the rbeta command in R. The alpha and beta values were derived from the estBetaParams function:

estBetaParams <- function (mu, var){

alpha <- ((1 - mu) / var - 1 / mu) * mu ^ 2

beta <- alpha * (1 / mu - 1)

return(params = list(alpha = alpha, beta = beta))

}
