# Supplementary material for: Epidemiological, clinical and radiological characteristics of people with neurocysticercosis in Tanzania–A cross-sectional study
Source: PLoS Negl Trop Dis. 2022 Nov 28;16(11):e0010911. doi: 10.1371/journal.pntd.0010911 (PMC9704569; doi:10.1371/journal.pntd.0010911)
Supplement: S4 Table — (DOCX) [file pntd.0010911.s006.docx]

S4 Table. Demographic and clinical characteristics of patients with mixed stage and inactive stage neurocysticercosis

|  |  | **Mixed stage** | **Inactive stage** | **p-value** |
| --- | --- | --- | --- | --- |
| N |  | 29 | 24 |  |
| **Demographics** |  |  |  |  |
| Site | Ifisi | 3 (10) | 6 (25) | 0.36 |
|  | Tukuyu | 8 (28) | 6 (25) |  |
|  | Vwawa | 18 (62) | 12 (50) |  |
| Sex | Female | 9 (31) | 16 (67) | 0.02 |
|  | Male | 20 (69) | 8 (33) |  |
| Age in years | Median [IQR] | 49 [32, 54] | 43 [26, 46.8] | 0.07 |
| Age group | (0,20] | 0 | 1 (4) | 0.71 |
|  | (20,40] | 11 (38) | 9 (38) |  |
|  | (40,60] | 16 (55) | 13 (54) |  |
|  | (60,80] | 2 (7) | 1 (4) |  |
| Religion | Christian | 26 (90) | 20 (83) | 0.79 |
|  | Muslim | 3 (10) | 4 (17) |  |
|  | Other or no religion | 3 (10) | 4 (17) |  |
| **Epileptic seizures** |  |  |  |  |
| Age of seizure onset | Median in years [IQR] | 29 [22, 42] | 28 [12, 31.5] | 0.13 |
| Time since seizure onset | Median in years [IQR] | 9 [8, 23] | 15 [6, 19.5] | 0.67 |
| Seizure frequency before treatment | Daily to monthly | 7 (24.1) | 10 (43.5) | 0.48 |
|  | Monthly to yearly | 17 (58.6) | 11 (47.8) |  |
|  | Yearly | 2 (6.9) | 1 (4.3) |  |
|  | Less than yearly, irregularly | 3 (10.3) | 1 (4.3) |  |
|  | Median per year [IQR] | 12 [4, 12] | 12 [12, 75] | 0.07 |
| Seizure frequency after treatment | Daily to monthly | 2 (6.9) | 0 (0.0) | 0.11 |
|  | Monthly to yearly | 14 (48.3) | 6 (26.1) |  |
|  | Yearly | 6 (20.7) | 5 (21.7) |  |
|  | Less than yearly, irregularly | 7 (24.1) | 12 (52.2) |  |
|  | Median [IQR] | 3 [1, 4] | 0.5 [0.2 ,2.3] | 0.04 |
| **Headache** |  |  |  |  |
| Frequent history of headache |  | 10 (35) | 8 (33) | 0.99 |
| Headache scale | 0 = Very happy, no hurt | 1 (10) | 0 | 0.71 |
|  | 1 = Hurts just a little bit | 1 (10) | 0 |  |
|  | 2 = Hurts a little more | 2 (20) | 2 (25) |  |
|  | 3 = Hurts even more | 3 (30) | 2 (25) |  |
|  | 4 = Hurts a whole lot | 3 (30) | 4 (50) |  |
| Headache quality | Pressure | 2 (20) | 1 (13) | 0.99 |
|  | Piercing | 0 | 1 (13) | 0.92 |
|  | Throbbing | 7 (70) | 8 (100) | 0.67 |
|  | Stabbing | 2 (20) | 0 | 0.56 |
| Headache duration | Up to one hour | 4 (40) | 5 (63) | 0.22 |
|  | Between one hour and one day | 3 (30) | 2 (25) |  |
|  | One day or longer | 3 (30) | 1 (13) |  |
| Headache frequency | Up to weekly | 5 (50) | 4 (50) | 0.99 |
|  | weekly to monthly | 5 (50) | 4 (50) |  |
|  | Less than once per month | 0 | 0 |  |
